# “Multivariate analysis of the impact of sleep and working hours on medical errors: a MICE approach"

**DOI:** 10.1186/s12889-023-17130-4

**Published:** 2023-11-23

**Authors:** Malena Lis Mul Fedele, María del Pilar López Gabeiras, Guido Simonelli, Joaquín José Diez, Giannina Julieta Bellone, Joaquín Cagliani, Luis Larrateguy, Kumiko Eiguchi, Diego Andrés Golombek, Daniel Pedro Cardinali, Daniel Pérez-Chada, Daniel Eduardo Vigo

**Affiliations:** 1 Chronophysiology Lab, Institute for Biomedical Research (UCA-CONICET), Buenos Aires, Argentina; 2 Austral University, Austral University Hospital, Pilar, Argentina; 3 Centre d’études Avancées en Médecine du Sommeil, Hôpital du Sacré-Coeur de Montréal, CIUSSS du Nord de L’Île-de-Montréal, Montreal, Canada; 4 Department of Medicine, Faculty of Medicine, University of Montreal, Montreal, Canada; 5 Department of Neuroscience, Faculty of Medicine, University of Montreal, Montreal, Canada; 6 Pan-American Institute of Sleep Medicine and Chronobiology, Buenos Aires, Argentina; 7 Pontifical Catholic University of Argentina, Buenos Aires, Argentina; 8 Chronobiology Lab, Department of Science and Technology, National University of Quilmes, Bernal, Argentina; 9 Anesthesiology Department, North Shore University Hospital, Manhasset, NY USA; 10 Private Center of Respiratory Medicine of Paraná, Entre Ríos, Argentina; 11 University of Salvador, Buenos Aires, Argentina; 12 Interdisciplinary Time Lab, San Andrés University, Buenos Aires, Argentina; 13 Katholieke Universiteit Leuven, Leuven, Belgium

**Keywords:** Sleep, Circadian rhythms, Resident physicians, Medical errors, Fatigue, Multiple imputation by chained equations (MICE)

## Abstract

**Background:**

The main objective of this study was to describe the relationship between working conditions, sleep and psycho-affective variables and medical errors.

**Methods:**

This was an observational, analytical and cross-sectional study in which 661 medical residents answered questionnaires about working conditions, sleep and psycho-affective variables. Actigraphic sleep parameters and peripheral temperature circadian rhythm were measured in a subgroup of 38 subjects. Bivariate and multivariate predictors of medical errors were assessed.

**Results:**

Medical residents reported working 66.2 ± 21.9 weekly hours. The longest continuous shift was of 28.4 ± 10.9 h. They reported sleeping 6.1 ± 1.6 h per day, with a sleep debt of 94 ± 129 min in workdays. A high percentage of them reported symptoms related to psycho-affective disorders. The longest continuous shift duration (OR = 1.03 [95% CI, 1.00–1.05], *p* = 0.01), working more than six monthly on-call shifts (OR = 1.87 [95% CI, 1.16–3.02], *p* = 0.01) and sleeping less than six hours per working day (OR = 1.66 [95% CI, 1.10–2.51], *p* = 0.02) were independently associated with self-reported medical errors. The report of medical errors was associated with an increase in the percentage of diurnal sleep (2.2% [95% CI, 0.1–4.3] vs 14.5% [95% CI, 5.9–23.0]; *p* = 0.01) in the actigraphic recording.

**Conclusions:**

Medical residents have a high working hour load that affect their sleep opportunities, circadian rhythms and psycho-affective health, which are also related to the report of medical errors. These results highlight the importance of implementing multidimensional strategies to improve medical trainees’ sleep and wellbeing, increasing in turn their own and patients’ safety.

**Supplementary Information:**

The online version contains supplementary material available at 10.1186/s12889-023-17130-4.

## Background

Modern society is subjected to different demands, like extended working hours and shift work, which can interfere with the homeostatic and circadian regulation of sleep with negative consequences in health and wellbeing [1]. In the long term, sleep deprivation and circadian disruption can cause serious health disorders, such as obesity [2], cancer [3] or cardiovascular diseases [4]. In the short term, one of the main consequences is fatigue, which, in this context, is defined as the state of sleepiness resulting from factors as time of the day, duration of wakefulness and quantity and quality of prior sleep [5]. Fatigue can significantly reduce performance, productivity, attention, vigilance, communication and manual skills and interfere with the ability to make decisions and to do complex planning; which in turn increase error rates, as well as the number of adverse incidents, accidents and injuries [6].

Medical residents are particularly affected because, along with the extended working hours and nocturnal work, they have an intense academic load, which includes training courses, research activities, and must devote time to study and get training in medical procedures. In turn, this contributes to increased stress levels, irregular sleep and circadian disruption [7, 8]. In fact, many studies have shown that, in resident physicians, sleep disorders are related with adverse outcomes, such as fatigue, motor vehicle crashes, percutaneous injuries and attentional failures [8–11].

Over the last decades, several countries have limited the in hospital hours for resident physicians in order to improve their health, increase their safety and decrease medical errors and adverse incidents [12–14]. However, there are still many controversies around this issue [15]. In some studies, medical errors were found to be related to extended shifts [16–18], while in others, the elimination of such extended shifts did not change or worsen its occurrence [19, 20]. It can be suggested that this contradiction arises because reducing medical errors is influenced not only by the length of the shift, but also by other factors such as the work schedule, supervision, and the number of patients seen per hour. These additional variables must also be considered. Therefore, when implementing duty hour restrictions, it is necessary to focus on many variables at the same time. A recent systematic review demonstrated that focusing on duty hours alone did not result in improvements in patients care or residents well-being and may have a negative effect on resident’s education [20]. This reflects the importance of studying the optimal combination of individual- and organizational-focused interventions.

Furthermore, these interventions must be tailored for different contexts, so it is necessary to carry out studies that encompass different realities. Regulations may vary locally, and in practice residents may work continuously for more than 30 h with only brief moments to rest or nap [21, 22]. Although there are some studies that explore Argentine resident physicians’ working hours [23, 24], little is known about their sleep–wake cycle and its impact on medical errors.

Thus, the first objective of this study was to describe working conditions, sleep and psycho-affective variables in a sample of Argentine medical residents. Furthermore, we used Multiple Imputation by Chain Equations (MICE) to analyze predictors of medical errors among residents, in order to address the problem of missing data and provide a more comprehensive understanding of the factors that contribute to medical errors in this population. In addition, we also studied a subgroup of residents to objectively assess their sleep–wake cycle and their temperature circadian rhythm. By doing so, this study aims to contribute to the ongoing efforts to reduce medical errors and improve patient safety in medical residency programs.

## Methods

### Study design and population

**Fig. 1:**
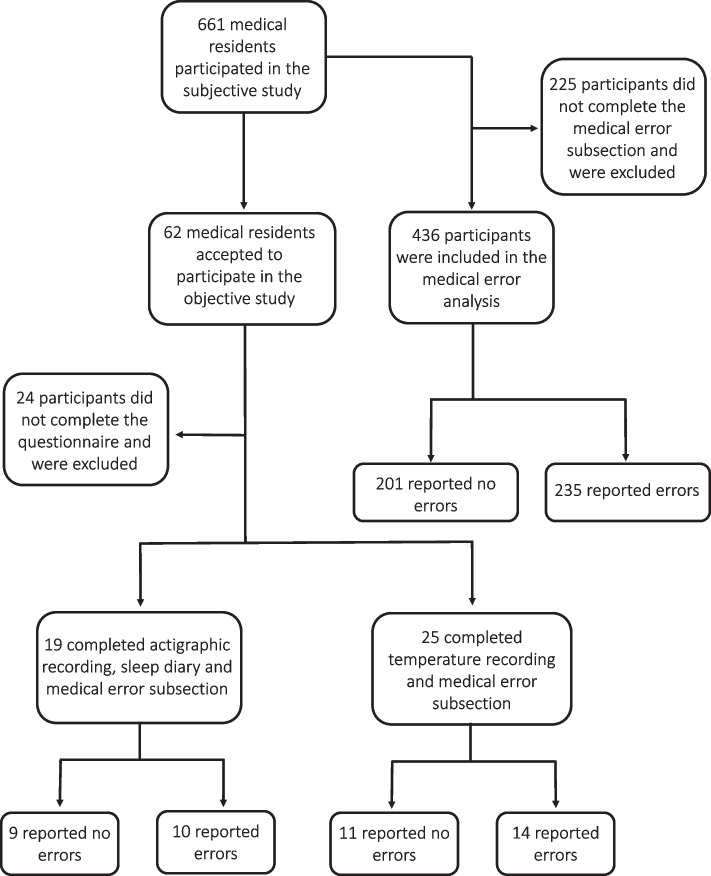
Schematic description of the study design. A total of 661 medical residents participated in the subjective study, but only 436 of them answered the medical error subsection and thus, were included in the medical error analysis. Of the participants, 62 accepted to take part in the objective analysis. However, 24 of them did not respond the questionnaire and were excluded. Only 19 of them had at least six complete days of actigraphy, completed the sleep log correctly and answered the medical error subsection. Additionally, 25 of them had almost three continuous days of temperature register and completed the medical error subsection

The aim of the study was to perform a multivariate analysis of an observational, analytical and cross-sectional exploratory study conducted between September 2011 and December 2012. Invitations were extended to all clinical and surgical residents from 19 Argentine hospitals (11 from the Autonomous City of Buenos Aires; one from Derqui, Buenos Aires Province; and seven from Paraná, Entre Ríos Province). Hospitals were selected based on a non-random convenience sampling approach. Response rate was estimated at 40%. In Argentina, medical residency programs mainly last four years and we included participants from all levels of training. As shown in Fig. 1, the study was conducted in an initial sample of 661 subjects. The participants who completed the medical error subsection were divided into two groups based on their medical error reporting: the non- medical error group (*n* = 201) and the medical error group (*n* = 235). A subset of 62 participants accepted the invitation to take part in the objective study. This modest participation rate can be understood in light of the same work overload factors examined in the current study. Out of these, only 38 participants completely responded the questionnaire. Among them, 19 individuals had successfully completed at least six full days of actigraphy along with accurate sleep log entries (non-medical error group, *n* = 9; medical error group, *n* = 10), while 25 participants maintained at least three consecutive days of temperature recording (non-medical error group, *n* = 11; medical error group, *n* = 14).

### Measurements

#### Questionnaires

Subjective variables were studied using different questionnaires that the participant completed alone. We used an adapted version of a survey designed by Jagsi et al. [25], translated to Spanish and tested for its comprehension. This questionnaire included clinical and demographic data. Overweight was defined as BMI ≥ 25, and obesity as BMI ≥ 30.

In the questionnaire, there was a section dedicated to adverse events, where the residents were asked to answer how many medical errors they had made in the last week. A medical error was explicitly defined in the questionnaire as any action or omission made by the medical resident that would be judged as incorrect by qualified professionals, and that could or could not cause complications, lesions or harm to the patient. The group that did not complete this subsection was excluded from the medical error analysis (Fig. 1). Differences between the group included and the group excluded in this analysis are shown in Supplementary Table 1.

The questionnaire also asked about some variables of their sleep–wake cycle. They were queried about the duration of their longest sleep period, the frequency and duration of their naps, and their perceived sleep requirement, both on workdays and on days off. The main sleep episodes were considered as anchor sleep and naps were considered as non-anchor sleep. Total sleep was the result from the sum of anchor and non-anchor sleep. Sleep debt was calculated as the difference between desired sleep and total sleep. We also calculated sleep debt between free and working days as the difference between total sleep on free days and total sleep on workdays. Sleep deprivation was defined as sleeping less than six hours per day.

Another section of the questionnaire pertained to working characteristics. In this section, participants were queried about the following aspects over the past four weeks: frequency of on-call shifts, the average duration of on-call shifts, the average number of hours of sleep during on-call shifts, and the number of days they experienced significant tiredness. Additionally, they were asked to provide information on the following parameters of the previous week: average number of patients attended daily, total on-duty hours, maximum uninterruptedly hours at work, minimum hours outside work between two consecutive workdays, total hours of sleep at work, total hours of sleep outside work, total hours devoted to minor procedures, total hours dedicated to major procedures, and total hours engaged in direct patient care.

A Spanish validation of the Pittsburgh Sleep Quality Index (PSQI) was used to study sleep quality. A score between 6 and 7 requires medical attention, a score between 8 and 14 requires medical attention and treatment, and a score higher than 15 is associated with serious sleep problems [26]. The Spanish validated version of the Epworth Sleepiness Scale (ESS) was used to measure daytime somnolence. An ESS score between 11 and 14 indicates mild daytime sleepiness, a score between 15 and 17 indicates moderate daytime sleepiness while a score higher than 18 indicates excessive daytime sleepiness symptoms [27]. Psycho-affective variables were studied using Spanish validations of the Beck Anxiety Inventory (BAI), the short form of the Beck Depression Inventory (BDI) and the Maslach Burnout Inventory (MBI; Copyright ©1981, 2016 by Christina Maslach & Susan E. Jackson. All rights reserved in all media. Published by Mind Garden, Inc., www.mindgarden.com). The BAI is a screening measure of frequency and intensity of anxiety symptoms, that contains 21 items on a 4-point Likert scale, with 0 representing 'not at all' and 3 being 'severely'. Scores of 0–7 are considered “minimal anxiety”; 8–15 are “mild anxiety”; 16–25 are “moderate anxiety”; and 26–63 are “severe anxiety” [28]. The BDI-short form is a scale used to study depression symptoms. It is a 13-item self-report questionnaire with a 4-point Likert scale ranging from 0 (not at all) to 3 (severely). The inventory classifies symptom severity in four categories: none or minimal (0–4 pts), mild (5–7 pts), moderate (8–15 pts) or severe (more than 16 pts) depression [29]. Burnout is known as the response to chronic emotional and interpersonal stressors on the job. MBI is used to assess burnout in 3 dimensions: Maslach Emotional Exhaustion score (MEE), Maslach Depersonalization score (MDP) and Maslach Personal Accomplishment score (MPA). Based on normative data, MEE ≥ 27, MDP ≥ 10 and MPA ≤ 33 are considered high scores [30].

#### Sleep–wake cycle

The sleep–wake cycle was objectively assessed using wrist accelerometers (Micro Motionlogger, Ambulatory Monitoring Inc., Ardsley, NY). Participants were asked to wear the devices in the nondominant wrist and to complete sleep logs during seven days. Sleep logs allowed to determine the sleeping location (home or hospital) and helped to visually identify “in bed” episodes in actigraphic recordings. The original sample was reduced to 19 individuals (Fig. 1) who were the ones that correctly completed the sleep log, had an actigraphic recording at least during six complete days and completed the medical error subsection of the questionnaire. Actigraphy data was analyzed using Action W 2.7 software (Ambulatory Monitoring Inc., Ardsley, NY). Reported sleep duration was standardized to a seven-day period by dividing sleep duration values by the total recording time and multiplying these results by seven. Periods of sleep starting between 8AM and 7PM and ending before 9PM were considered as diurnal sleep. As an indicator of sleep misalignment, we reported the “Composite Phase Deviation” index. For each day, it is calculated as the square root of the sum of two squared values: the distance of mid-sleep on a given day to an individual reference (we used the acrophase of the circadian temperature rhythm), and the distance of midsleep on the given day to the previous day [31]. Sleep bouts shorter than 3 h were excluded from this analysis. If a sleep bout was shorter than 3 h, it was included as main sleep if it was the only one within 24 h or if it had similar sleep onsets (night shift) or offsets (morning shift) as the previous sleep bout in a block of consecutive shift. When more than one bout appeared within eight hours, mid-sleep was calculated as an average between the sleep onset of the first one and the sleep offset of the last one.

### Data analysis

Numerical variables in the descriptive analysis are shown as mean ± standard deviation (SD), and categorical variables as frequency (%). Differences between groups of numerical variables were assessed using a t-test for independent samples or a chi-square test in the case of categorical variables (Fisher test if expected frequencies were < 5). Bivariated correlations were analyzed with a Pearson correlation test.

A multivariate analysis was conducted to identify independent predictors of medical errors in the whole sample. Missing values (< 4% with a random pattern) were imputed using Multivariate Imputation via Chained Equations (MICE) package in R software. MICE is a general approach for imputing multivariate data, which replaces missing values with plausible values drawn from a distribution specifically modeled for each missing entry. The function generates an m number of imputed datasets (m = 5 in this study) which differ in the imputed values. The magnitude of these differences reflect the uncertainty in the imputed values [32]. Then, a binary logistic regression was conducted using the data of all the imputed datasets. We conducted different models including age, year of residence program, number of patients per day, specialty, total weekly hours on duty, longest continuous shift, monthly on-call shifts, anchor sleep, non-anchor sleep, sleep debt and sleep debt between free and working days, as independent predictors, and we chose the model that best adjusted to the data. Goodness of fit was assessed using the pooled C-statistic (area under the ROC curve), and the pooled Nagelkerke's R^2^ score.

Variables derived from actigraphic analysis are shown as mean with 95% CI. Normality was tested using the Kolmogorov–Smirnov test, and groups were compared by means of an unpaired t-test with Welch’s correction for independent samples.

### Ethical aspects

All subjects received detailed information about the procedures and gave written informed consent to participate before the study. The study was approved by the institutional review board of Universidad Austral (Derqui, Argentina) and was performed in accordance with the Declaration of Helsinki and its amendments.

## Results

### Sociodemographic and working characteristics

Young resident physicians composed the sample, with a mean age of 28.2 ± 2.6 years (Table 1), 69% were females. Mean body mass index was 22.6 ± 3.3 kg/m^2 ^with 21% showing overweight and 4% obesity.

**Table 1:** Demographic, working, sleep and psycho-affective characteristics

*Variable*	*Valid n*	*Mean/n*	*SD/%*
Mean age	657	28.2	2.6
Year of residency program	661	2.3	1.1
% following internal medicine specialty	661	500	75,6
Total weekly hours on duty in hospital	566	66.2	21.9
Longest continuous shift (hs)	583	28.4	10.9
Minimum hours outside work between two days	546	11.2	3.0
Weekly hours performing minor procedures	506	3.2	4.3
Weekly hours performing major procedures	399	3.7	7.9
Weekly hours of direct patient care	560	22.9	17.0
Number of patients per day	516	18	15
Monthly on-call shifts	614	6.1	3.0
Hours of active call	564	25.1	6.8
Days of significant fatigue during last month	603	13	8
% working > 80 hs in a week	566	158	27.9
% working > 30 hs continuosly	583	329	56.4
Weekly hours of sleep at work	544	4.7	3.9
Weekly hours of sleep outside work	421	34.2	8.5
Weekly total hours of sleep	362	39.1	8.9
Hours of sleep during on-call shift	548	3.0	1.6
Typical anchor sleep (hs) in workdays	654	6.1	1.6
Desired sleep duration (hs)	641	8.0	1.3
Typical naps (n°) in workdays	167	1.6	0.9
Typical nap duration (min) in workdays	165	60	38
Non-anchor sleep (min) in workdays	661	23	62
Total sleep (hs) in workdays	654	6.5	1.9
Sleep debt (min) in workdays	641	94	129
Typical anchor sleep (hs) in free days	646	9.3	2.0
Desired sleep duration (hs) in free days	619	8.9	1.9
Typical naps (n°) in free days	413	1.3	0.7
Typical nap duration (min) in free days	400	84	46
Non-anchor sleep (min) in free days	661	66	87
Total sleep (hs) in free days	646	10.4	2.7
Sleep debt free – work days (min)	643	237	185
Pittsburgh Sleep Quality Index score	661	11.7	2.2
Epworth Sleepiness Scale score	661	14.2	4.9
% of sleep deprivated in workdays	654	163	24.9
% with a sleep debt > 2hs in workdays	641	173	27
% sleep deprivated in free days	646	11	1.7
% with a sleep debt > 2 hs in free days	613	28	1.6
% with a sleep debt free – work days > 2 hs	643	448	69,7
% with Pittsburgh Sleep Quality Index >6 and ≤7	661	16	2.4
% with Pittsburgh Sleep Quality Index >8 and ≤14	661	588	89
% with Pittsburgh Sleep Quality Index >15 and ≤21	661	55	8.3
% with Epworth Sleepiness Scale >0 and ≤10	661	147	22.3
% with Epworth Sleepiness Scale >11 and ≤14	661	174	26.4
% with Epworth Sleepiness Scale >15 and ≤17	661	154	23.3
% with Epworth Sleepiness Scale >18 and ≤24	661	185	28
Beck Anxiety Inventory score	661	9.6	7.9
Beck Depression Inventory score	661	5.1	4.1
Maslach Emotional Exhaustion score	661	28.2	11.8
Maslach Depersonalization score	661	9.8	7.3
Maslach Personal Acomplishment score	661	34.8	9.5
% with Beck Anxiety Inventory score <8	661	323	48.9
% with Beck Anxiety Inventory score >8 and ≤15	661	212	32.1
% with Beck Anxiety Inventory score >16 and ≤25	661	101	15.3
% with Beck Depression Inventory score <5	661	340	51.4
% with Beck Depression Inventory score >5 and ≤7	661	168	25.4
% with Beck Depression Inventory score >8 and ≤15	661	141	21.3
% with Maslach Emotional Exhaustion score ≥27	661	376	56.9
% with Maslach Depersonalization score ≥ 10	661	323	48.9
% with Maslach Personal Acomplishment score ≤ 33	661	233	35.2

Total *n* was 661

Mean is reported for numerical and percentage for categorical data

Working characteristics of the sample are shown in Table 1. Most of them (46.1%) were on their first year of the residence program, 22.4% in the second year and 19% in the third year (data not shown). 76% were internal medicine residents, 15% surgery residents and 10% did not report their training program. Residents reported working an average of 66.2 ± 21.9 weekly hours with a longest continuous shift of 28.4 ± 10.9 hs. The minimum hours outside work between two shifts was of 11.2 ± 3.0 hs. Moreover, 28% reported working more than 80 hs a week and a 56% working more than 30 hs continuously, with around 6 monthly on-call shifts on average.

### Sleep habits and psycho-affective variables

The subjective analysis of sleep habits and psycho-affective variables is shown in Table 1. Residents reported sleeping a mean of 4.7 ± 3.9 weekly hours at work, 34.2 ± 8.5 weekly hours outside work and 3.0 ± 1.6 hs during the extended shift. Typical anchor sleep on workdays was of 6.1 ± 1.6 hs on average and approximately 25% of the sample reported taking naps on workdays, with a mean duration of 60 ± 38 min. Sleep debt in workdays was of 94 ± 129 min, 25% of the residents slept less than 6 hs per day, and 27% showed a sleep debt of more than 2 hs.

During free days, residents reported sleeping 9.3 ± 2.0 hs on average, and approximately 63% reported taking naps with a mean duration of 84 ± 46 min. Sleep debt between free and workdays, was of 237 ± 185 min on average, with 70% of the sample showing a sleep debt of more than 2 hs.

Mean score for PSQI was of 11.7 ± 2.2; 89% of the sample reported a score between 8 and 14 (symptoms that require medical attention and treatment) and 8% greater or equal to 15 (severe sleep problems). The mean ESS score was of 14.2 ± 4.9; 23% reported scores between 15 and 17 (symptoms of moderate sleepiness), and 28% reported a score greater or equal to 11 (symptoms of excessive sleepiness).

Regarding psycho-affective variables, 15% of the sample reported symptoms related to moderate anxiety (BAI scale), 21% symptoms related to moderate depression (BDI scale), 57% symptoms related to emotional exhaustion, 49% related to depersonalization, and 35% felt low personal accomplishment.

Bivariate correlations between working, sleep and psycho-affective variables are shown in supplementary Table 2. In general, extensive working hours were associated with short sleep duration, bad sleep quality, depression and burnout.

### Bivariate and multivariate predictors of medical errors

Resident physicians also reported about medical errors in the last week; 34% did not complete this questionnaire subsection and 35.6% reported a medical error. The group that did not complete this subsection was excluded and differences with the group that completed it are shown in supplementary Table 1. The excluded group was 7 months older and reported having a longest continuous shift 2.6 hs shorter. They also obtained a lower score in the Maslach Emotional Exhaustion and Depersonalization scales.

Demographic and working characteristics were studied according to reported medical errors and results are shown in Table 2. Participants who reported medical errors worked on average 7.4 h more per week (*p* = 0.001), had a longest continuous shift approximately 3 hs longer (*p* = 0.003), reported more days experiencing significant fatigue (*p* = 0.004), and had about one more monthly on-call shift (*p* < 0.001), than the group who reported no medical errors. Also, a higher percentage of those who reported medical errors worked more or equal than 80 weekly hours (22% vs 36%; *p* = 0.004) and more than 30 hs continuously (54% vs 65%; *p* = 0.04), than those who didn`t.

**Table 2:** Demographic, working, sleep and psycho-affective characteristics according to reported medical errors

*Variable*	*Medical errors = 0*			*Medical errors ≥ 1*			*Test*
	*Valid n*	*Mean/n*	*SD/%*	*Valid n*	*Mean/n*	*SD/%*	*p*
Mean age	200	28.3	2.3	233	27.8	2.2	0.05
Year of residency program	201	2.5	1.2	235	2.3	1.0	0.22
% following internal medicine specialty	189	162	85.5	221	180	81.4	0.25
Total weekly hours on duty in hospital	177	62.7	21.1	207	70.1	21.1	^a^**0.001**
Longest continuous shift (hs)	180	27.6	11.3	209	30.7	9.1	^a^**0.003**
Minimum hours outside work between two days	171	11.4	3.1	207	10.9	2.9	0.09
Weekly hours of performing minor procedures	163	3.0	3.5	203	3.7	4.9	0.09
Weekly hours of performing major procedures	135	2.4	5.8	150	3.8	8.0	0.09
Weekly hours of direct patient care	176	22.2	16.1	207	23.2	16.2	0.54
Days of significant fatigue during last month	183	12.1	7.8	222	14.3	7.3	^a^**0.004**
Monthly on-call shifts	190	5.3	3.0	224	6.6	3.0	^a^**< 0.001**
Hours of active call	163	24.9	6.9	214	25.8	6.3	0.17
Number of patients per day	211	14.2	12.9	237	15.5	14.7	0.33
% working ≥ 80 hs	177	38	21.5	207	72	34.8	^a^**0.004**
% working ≥ 30 hs continuosly	180	98	54.4	209	135	64.6	^a^**0.04**
Weekly hours of sleep at work	169	4.2	3.7	212	5.0	4.1	^a^**0.04**
Weekly hours of sleep outside work	122	34.7	8.2	163	33.9	8.6	0.44
Weekly total hours of sleep	106	39.3	8.8	148	38.9	9.2	0.74
Hours of sleep during on-call shift	159	3.0	1.7	209	2.9	1.5	0.42
Typical anchor sleep (hs) in workdays	201	6.3	1.6	232	5.9	1.5	^a^**0.007**
Desired sleep duration (hs)	198	8.1	1.5	227	8.0	1.2	0.89
Typical naps (n°) in a work day	47	1.6	0.9	57	1.5	0.8	0.45
Typical nap duration (min) in workdays	44	60	34	59	57	42	0.68
Non-anchor sleep (min) in workdays	201	24	70	232	19	45	0.41
Total sleep (hs) in workdays	201	6.7	1.9	232	6.2	1.8	^a^**0.006**
Sleep debt (min) in workdays	198	83	127	227	111	118	^a^**0.02**
Typical anchor sleep (hs) in free days	200	9.3	2.0	226	9.4	2.1	0.61
Desired sleep duration (hs) in free days	191	9.0	2.1	222	9.0	2.0	0.83
Typical naps (n°) in free days	123	1.3	0.6	154	1.3	0.7	0.97
Typical nap duration (min) in free days	118	85	42	150	84	47	0.85
Non-anchor sleep (min) in free days	201	65	88	235	69	88	0.57
Total sleep (hs) in free days	200	10.4	2.9	226	10.5	2.8	0.52
Sleep debt free – workdays (min)	200	221	183	224	260	183	^a^**0.03**
Pittsburgh Sleep Quality Index score	201	11.2	2.1	235	12.2	2.1	^a^**< 0.001**
Epworth Sleepiness Scale score	201	13.8	4.8	235	15.0	4.9	^a^**0.01**
% of sleep deprivated in workdays	201	47	23.4	232	70	30.2	0.11
% with a sleep debt > 2hs in workdays	198	47	23.7	227	71	31.3	0.08
% sleep deprivated in free days	200	3	1.5	226	6	2.7	0.51
% with a sleep debt > 2 hs in free days	191	12	6.3	217	9	4.1	0.33
% with a sleep debt free – work days > 2 hs	200	130	65.0	224	168	75.0	^a^**0.02**
% with 6 < Pittsburgh Sleep Quality Index > 7	201	5	2.5	235	2	0.9	0.912
% with 8 < Pittsburgh Sleep Quality Index > 14	201	184	91.5	235	207	88.1	0.175
% with 15 < Pittsburgh Sleep Quality Index > 21	201	11	5.5	235	25	10.6	0.237
% with 0 < Epworth Sleepiness Scale > 10	201	53	26.4	235	42	17.9	^a^**0.032**
% with 11 < Epworth Sleepiness Scale > 14	201	57	28.4	235	54	23.1	0.199
% with 15 < Epworth Sleepiness Scale > 17	201	44	21.9	235	59	25.2	0.431
% with 18 < Epworth Sleepiness Scale > 24	201	47	23.4	235	79	33.8	^a^**0.019**
Beck Anxiety Inventory score	201	9.0	8.1	235	10.6	7.8	^a^**0.04**
Beck Depression Inventory score	201	4.6	3.6	235	5.7	4.3	^a^**0.005**
Maslach Emotional Exhaustion score	201	27.4	12.2	235	30.9	10.8	^a^**0.002**
Maslach Depersonalization score	201	9.3	7.1	235	11.5	7.2	^a^**0.002**
Maslach Personal Acomplishment score	201	35.4	9.0	235	34.9	8.3	0.55
% with 8 < Beck Anxiety Inventory score > 15	201	56	27.9	235	85	36.2	0.07
% with 16 < Beck Anxiety Inventory score > 25	201	27	13.4	235	40	17.0	0.30
% with Beck Anxiety Inventory score > 26	201	7	3.5	235	12	5.1	0.41
% with 5 < Beck Depression Inventory score > 7	201	55	27.4	235	65	27.7	0.95
% with 8 < Beck Depression Inventory score > 15	201	32	15.9	235	62	26.4	^a^**0.008**
% with Beck Depression Inventory score > 16	201	2	1.0	235	6	2.6	0.30
% with Maslach Emotional Exhaustion score ≥ 27	201	109	54.2	235	157	66.8	^a^**0.007**
% with Maslach Depersonalization score ≥ 10	201	91	45.3	235	137	58.3	^a^**0.007**
% with Maslach Personal Acomplishment score < 33	201	132	65.7	235	154	65.5	0.98

Mean is reported for numerical and percentage for categorical data

^a^Significant difference in the independent sample t-test (for numerical variables) or in the Chi-square test (for categorical variables)

We also assessed sleep habits according to reported medical errors (Table 2). The group that informed medical errors reported sleeping about 50 min more at the hospital (*p* = 0.04), having a typical anchor sleep 25 min shorter per working day (*p* = 0.007), sleeping 30 min less per working day (*p* = 0.006), having a 30 min greater sleep debt in workdays (*p* = 0.02), and having a sleep debt between work and free days approximately 40 min higher (*p* = 0.03), than the group that reported no medical errors. Furthermore, a greater percentage of the subjects who reported medical errors had a sleep debt of more than 2 hs between free and working days (75% vs 65%; *p* = 0.02). Regarding sleep scales, a greater percentage of those who reported medical errors, reported symptoms of excessive sleepiness (23% vs 34%; *p =* 0.04).

Regarding bivariate psycho-affective predictors (Table 2), a higher percentage of the group that reported medical errors reported symptoms related to moderate depression (26% vs 16%; *p* = 0.008), to emotional exhaustion (67% vs 54%; *p* = 0.007) and to depersonalization (58% vs 45%; *p* = 0.007).

In the multivariate analysis shown in Table 3 (C-statistic = 0.66 [95% CI, 0.61–0.71]; Nagelkerke's R^2^ = 0.10), the longest continuous shift duration (OR = 1.03 [95% CI, 1.00–1.05], *p* = 0.01), working more than six monthly on-call shifts (OR = 1.87 [95% CI, 1.16–3.02], *p* = 0.01) and sleeping less than six hours per working day (OR = 1.66 [95% CI, 1.10–2.51], *p* = 0.02), were independently associated with self-reported medical errors.

**Table 3:** Binary logistic regression of the relationship between working and sleep characteristics with reported medical errors

Variable	Statistic	Odds Ratio (95% CI)	*p*
**Year of residency program > 2**	0.37	1.10 (0.68-1.75)	0.71
**Age**	-0.77	0.97 (0.88-1.05)	0.44
**Number of patients per day**	1.24	1.01 (0.99-1.03)	0.22
**Surgery specialty**	-0.57	0.83 (0.43-1.61)	0.57
**Total weekly hours on duty > 80 hours**	0.22	1.06 (0.63-1.77)	0.82
**Longest continuous shift duration (hs)**	2.46	1.03 (1.00-1.05)	*0.01
**Monthly on-call shifts > 6**	2.57	1.87 (1.16-3.02)	*0.01
**Anchor sleep ≤ 6 hours in work days**	2.40	1.66 (1.10-2.51)	*0.02
**Sleep debt between work and free days > 3 hours**	-0.18	0.96 (0.64-1.45)	0.85

*Significantly different

### Objective analysis of the sleep–wake cycle and the temperature circadian rhythm

Sociodemographic characteristics of the subsample that participated in the objective study are shown in Supplementary Table 3.

**Fig. 2:**
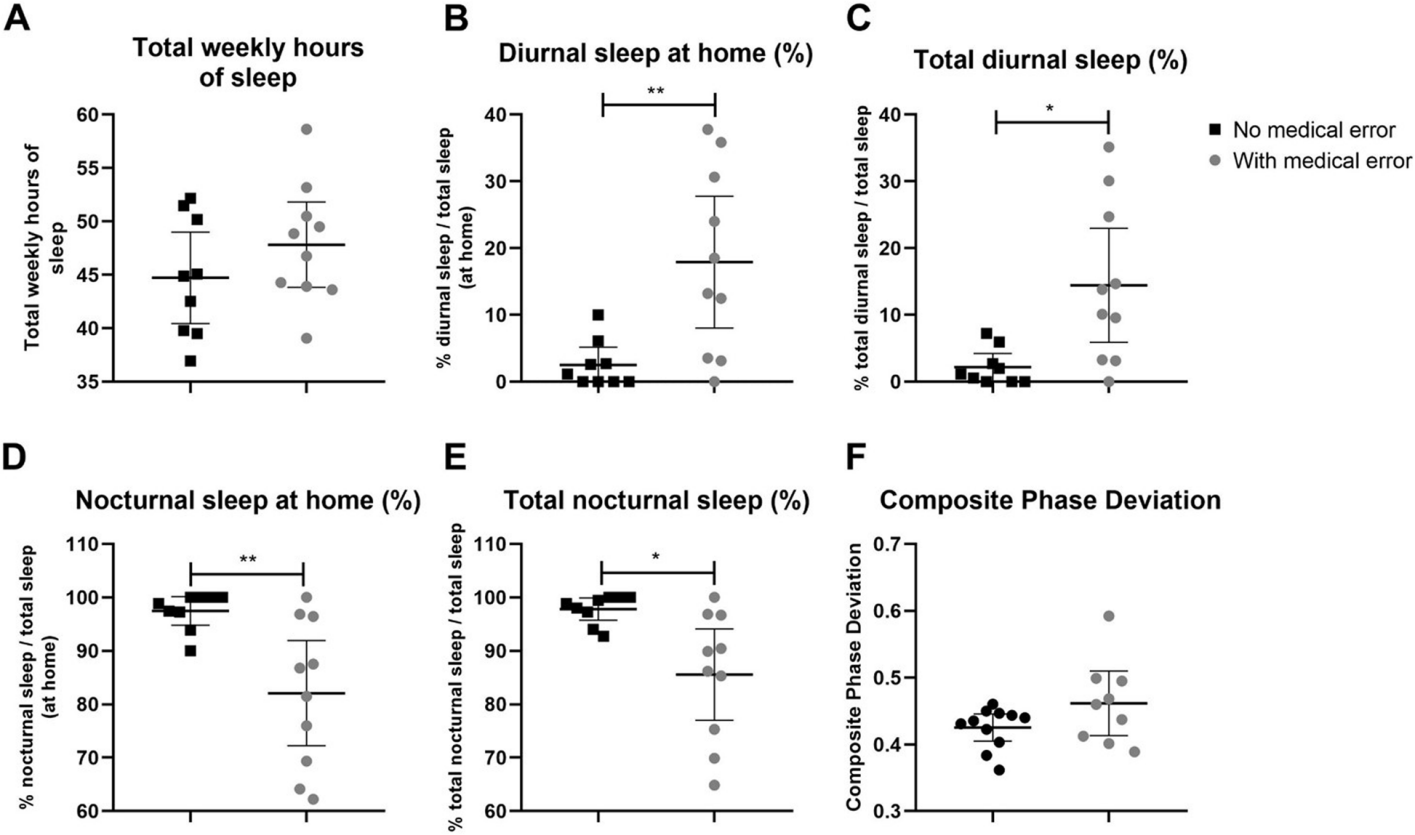
Sleep characteristics of the medical residents according to the actigraphic data. Shown are mean with 95% CI of: **A**) total weekly hours of sleep, **B**) percentage of diurnal sleep at home, **C**) percentage of total diurnal sleep, **D**) percentage of nocturnal sleep at home, **E**) percentage of total nocturnal sleep and **F**) Composite Phase Deviation index (No medical error group: *n* = 9; Medical error group: *n* = 10). Unpaired t-test with Welch’s correction: B) **p = 0.006, C) *p = 0.01, D) **p = 0.006 and E) *p = 0.01

A summary of actigraphic data is shown in Fig. 2. We found that resident physicians who reported medical errors had a greater percentage of diurnal sleep than those who did not (Fig. 2-C; No medical error group, 2.2% [95% CI, 0.1–4.3]; Medical error group, 14.5% [95% CI, 5.9–23.0]; *p* = 0.01). In turn, the sub-group reporting medical errors exhibited significantly less nocturnal sleep (Fig. 2-E; No medical error group, 97.8% [95% CI, 95.7–99.9]; Medical error group, 85.6% [95% CI, 77.0–94.1]; *p* = 0.01). No differences between groups were found when analyzing circadian misalignment (composite phase deviation index).

When we analyzed bivariate correlations (Supplementary Table 4), we found that the percentage of diurnal sleep positively correlated with MEE, MDP and BAI scores; and that total weekly hours of extended shift positively correlated with the percentage of diurnal sleep, ESS, MEE and BAI scores.

Finally, regarding the temperature rhythm, we found marginal no significant differences between groups in the amplitude (*p* = 0.09; Supplementary Fig. 1).

## Discussion

The main results of this study reveal that resident physicians in Argentina have a high workload, with twenty-eight percent of them working more than 80 hs per week and 56% of them working more than 30 hs continuously. They reported an anchor sleep on workdays of 6.1 hs on average, with 25% of them sleeping less than 6 hs per day. Moreover, we found that participants who reported medical errors worked more hours per week, had a longer longest-continuous shift, attended more monthly on-call shifts and slept 30 min less per working day, when compared with the group who reported no medical errors. Furthermore, a higher percentage of the sample that reported medical errors reported symptoms related to moderate depression, emotional exhaustion and depersonalization. We also found that the duration of the longest continuous shift, working more than six monthly on-call shifts and sleeping less than six hours per working day, are independent predictors of self-reported medical errors. Finally, in the objective study, we found that the participants that reported medical errors had a greater percentage of diurnal sleep, as well as reduced nocturnal sleep.

### Work, sleep and psycho-affective characteristics

Residents reported a weekly hour load of 66 hs on average. There was a high percentage of subjects (28%), who reported working more than 80 hs per week. Studies from other countries have found similar workloads in resident physicians [8, 13, 17]. In the last years, some countries established a restriction in resident physicians’ workload. In the United States maximum weekly working time was limited to 80 hs and duty period length must not exceed 24 hs for second-year residents [33]. In New Zealand, they are restricted to work not more than 72 hs per week and not more than 16 hs per day [34], while in Europe the average weekly working time must not exceed 48 hs with a daily hour rest period of 11 consecutive hours [35, 36]. According to the national normative in Argentina, resident physicians must work up to nine hours per day from Monday to Friday and perform a maximum of eight monthly on-call shifts of up to twelve hours, with a minimum rest of six hours after these extended shifts [37]. Therefore, regarding Argentina, our study shows that the local regulations are often not enforced. Nowadays, these regulations are being discussed, as some authors argue that work hour limitations can negatively affect resident’s education, the acquisition of professional skills, experience in the operating theater, and productivity [20, 36]. However, long working hours are associated with burnout, mental health, and deteriorated wellbeing and safety among resident physicians [7–11, 38]. In addition, many studies have shown that the reduction in working hours not only improves residents’ quality of life but also their quality of attention and performance [16, 39].

In our study, participants reported an anchor sleep of 6.1 hs in a typical working day, which is almost one hour less than the recommended sleep duration for a young adult [40], with 25% of the trainees sleeping less than six hours. The sleep duration observed in the objective study was quite longer (about 30 min more than in the subjective study), but still below the recommended seven hours of sleep per night. Studies from other countries have found similar results [21, 41]. It has been shown that chronic sleep restriction of six hours per night or less generates a deficit on cognitive performance equivalent to up to two nights of total sleep deprivation, generating a neurobiological “cost” that accumulates over time and that can seriously impair waking neurobehavioral functions, supporting the idea that humans cannot adapt to chronic sleep restriction [42]. During on-call nights, residents reported sleeping an average of three hours. This result is also similar to the one previously reported [21]. This amount of sleep is much less than the one required to perform adequately, especially considering that sleep in hospital is of lower quality than at home [43]. During extended shifts, interruptions of sleep not only reduce the overall amount of sleep, but also can lead to “sleep inertia” which can impair performance and has been shown to be more pronounced in sleep-deprived people [44]. It is possible that residents compensate this sleep loss with diurnal naps during the day after an extended shift. In our study, considering naps, sleep debt is practically reduced to zero, but we must consider that only 25% of the participants usually take naps. Basner and colleagues observed that sleep loss during the on-call night was partially recovered by interns through naps on the first post-call day. However, to fully recover sleep loss, they should have obtained more than four hours of sleep during the on-call night [21]. Something similar may be happening to the residents in our study, which could explain the increased percentage of participants who take naps during their free days.

The bivariate correlation analyses done in our study show that both the number of monthly on-call shifts and the length of the longest continuous shift negatively correlated with the amount of obtained sleep and positively correlated with sleep deprivation on workdays. So, as other studies have shown [21], extended overnight shifts increase the likelihood of chronic sleep deprivation in resident physicians. We can hypothesize that educational sleep hygiene programs are needed to increase awareness in this population. However, some studies have shown that these type of programs have no benefit on resident’s sleep [45, 46], possibly because medical residents are aware about the importance of sleeping and avoiding fatigue, but they do not have the necessary resting opportunities.

In this study, a high percentage of residents reported symptoms related to anxiety (47%), and depression (47%), along with burnout (57% emotional exhaustion, 49% depersonalization and 35% low personal accomplishment). Moreover, these variables positively correlated with workload. Several other studies showed that total hours of work are associated with burnout and wellness [47–49]. However, other characteristics of the medical profession, such as sleep deprivation, must be considered. In our study, we have shown that anchor sleep, diurnal sleep and sleep debt correlated with symptoms of anxiety, depression and Maslach scales scores. This adds to the evidence of studies reporting that sleep hours are negatively correlated with increased levels of stress [50].

### Univariate and multivariate predictors of medical errors

We found that total hours on duty, the length of the longest continuous shift and the number of on-call shifts were associated with reported medical errors. It is known that fatigue affects resident physicians cognitive performance and coordination, among other variables [50, 51]. A previous study done in Argentina has shown that orthopedic residents’ attention levels are affected after an extended shift of 24 h [23]. Our results show that sleep habits, such as the typical anchor sleep, total sleep and sleep debt in workdays, the sleep debt between work and free days, and the auto perceived quality of sleep and diurnal somnolence are also related to reported medical errors, as well as diurnal sleep objectively assessed. Some studies have shown that sleep deprivation after an extended shift adversely affects surgical residents’ performance on a simulator trial [52, 53] while others showed no significant effects [54]. Other groups have also found that extended-duration work shifts, high hourly load and night work were associated not only with an increased risk of medical errors, adverse events, and attentional failures, but also with personal injuries, accidents, and conflicts with other staff members [8, 10, 16, 55].

As previously mentioned, sleep deprivation and long working hours can also affect psycho-affective variables [56, 57]. In this study, we observed that anxiety, depression, and depersonalization scores were related to reported medical errors. These results are in line with those of previous studies that have found a relationship between burnout and residents’ self-reported errors [58].

The decline in shift worker’s performance is attributed to the misalignment caused by working and sleeping at the wrong circadian phase [59]. This discrepancy between the social and biological clocks is known as social jet lag [60]. Although the composite phase deviation index used in this study was not associated with medical errors, we observed a link between higher percentages of diurnal sleep and lower levels of nocturnal sleep and medical errors. Even though actigraphic data has an important dispersion, results from the objective study are consistent with those derived from the subjective study. Moreover, these findings align with the documented effects of extended shifts on both sleep and performance, as indicated in previous studies [52, 53, 55]. Indeed, the values of the composite phase deviation index found in this study are close to those reported in a study in long-haul bus drivers, who are subjected to high fatigue risk work schedules [61]. In turn, circadian disruption can worsen residents’ physical and mental health, wellbeing and performance [59, 62].

In the multivariate analysis reported in this study, we observed that the longest continuous shift, working more than six monthly on-call shifts and sleeping less than six hours per day were predictors of self-reported medical errors. It seems obvious that those who work more hours have a lower sleep opportunity. In line with this, there are other publications that show that sleep hours are negatively correlated with significant medical errors, and working in an impaired condition [50]. McCormick and colleagues found that residents’ fatigue levels increase the risk of medical errors by 22% [41]. Interestingly, Van Dongen et al. also have shown that neurobehavioral deficits were primarily caused by excessive wakefulness beyond a maximum period both across days of chronic sleep restriction and across days of total sleep deprivation [42]. Excessive wakefulness is a result of extended shifts and on-call nights, and, of course, it can be considered the main cause for sleep deprivation. Indeed, we could argue that in this population more attention must be paid to working hours policies than to sleep hygiene programs. In fact, a study found that the implementation of an sleep hygiene program had no significant effect on sleep deprivation in medical residents [45].

Finally, some limitations of this study must be considered. First, residents who reported higher values of working hours and burnout indexes may be more prone to show the impact of fatigue in their performance, determining recall bias. This may be associated with additional selection bias, since the residents who answered the medical error subsection showed higher reported values of extended working hours, exhaustion, and depersonalization. Second, even if the number of selected hospitals was high, the utilization of a non-random convenience approach introduces a potential limitation by compromising the results' generalizability to the entire country. Third, the findings from the objective study would have been more representative and conclusive if it had been done with a higher number of participants. Unfortunately, the compliance of medical residents to wear the actigraphs and temperature sensors during a 7-day period, and to fully complete the sleep log and questionnaire, was poor. Only 19 out of 38 recordings were valuable for actigraphic analysis, and 25 out of  38 for temperature rhythm analysis.

In addition to these limitations, this study also boasts several strengths. Firstly, a limited number of studies have undertaken the analysis of the relationship between sleep and medical errors by employing an approach that combines a multivariate analysis of subjective data with measures of objective parameters. Furthermore, to our best knowledge, no studies in this area conducted a multivariate analysis using MICE to handle missing data, a relatively novel tool with increasing use in epidemiological research [32, 63]. Finally, as previously mentioned, while the objective study’s sample size may be less than optimal, the subjective study encompasses a substantial number of participants, affording us to derive several conclusions.

Some recommendations warrant consideration for future research. Firstly, there is a need to develop new tools, while also validating existing tests, such as reaction time tasks or neuropsychological evaluations, for the detection of fatigue and performance impairment in healthcare settings. Additionally, a notable gap exists in the literature concerning research on the integration of strategic napping within hospital environments. Lastly, is essential to conduct research on the effects of implementing a Fatigue Risk Management System (FRMS) that considers multiple strategies to prevent and detect fatigue and burn out. Studies should encompass an evaluation of outcomes related to sleep patterns, fatigue levels, performance, and overall well-being of medical residents.

## Conclusions

Our study has shown that high workload, which can disrupt sleep patterns, is closely related to medical errors. Additionally, we have highlighted the importance of taking into account burn out and psycho-affective variables. These results highlight the importance of implementing appropriate shift-work scheduling strategies, identifying effective tools to monitor sleep and fatigue, and developing interventions to ameliorate the impact of circadian shift work disorders in medical residents’ health. As it was shown in other studies, different interventions, such as melatonin intake, strategic napping, and biomathemathical modeling of fatigue [64–66], could prevent sleep deprivation and circadian misalignment in resident physicians. However, an implementation alone is not sufficient to prevent and control fatigue.

Strategies should consider different variables concurrently. A potential approach could be the implementation of a FRMS for healthcare personnel, consisting of defensive layers aimed at preventing fatigue-related errors and incidents. These layers include the development of work schedules based on biomathematical fatigue modelling, the assessment of sleep duration, and the evaluation of signs of fatigue, especially during the vulnerable nighttime hours [67]. Additionally, the integration of planned naps in comfortable environments, and stress management techniques, such as mindfulness, would contribute to the mitigation of fatigue and the enhancement of residents' well-being. Finally, an increase in the number of medical residency positions to promote a more balanced distribution of workload among residents should complement this approach.

### Supplementary Information

**Additional file 1:** **Supplementary Figure 1.** Characteristics of the temperature circadian rhythm of medical residents. Mean with 95% CI of the A) amplitude, B) percentage of rhythmicity, C) mesor and D) acrophase of the temperature rhythm (No medical error group: *n*=11; Medical error group: *n*=14). **Supplementary table 1.** Characteristics of the participants that did not complete the medical error subsection (excluded). **Supplementary table 2.** Bivariate correlation between working characteristics, sleep habits and psyco-affective variables. **Supplementary table 3.** Demographic, working, sleep and psycho-affective characteristics of the objective study sample. **Supplementary table 4.** Bivariate correlation between objective working and sleep characteristics, and sleep and psyco-affective scales.

## Data Availability

The datasets used and/or analyzed during the current study are available from the corresponding author on reasonable request.
