# Supplementary material for: “Multivariate analysis of the impact of sleep and working hours on medical errors: a MICE approach"
Source: BMC Public Health. 2023 Nov 23;23:2317. doi: 10.1186/s12889-023-17130-4 (PMC10666331; doi:10.1186/s12889-023-17130-4)
Supplement: Supplementary file 1 — Additional file 1: Supplementary Figure 1. Characteristics of the temperature circadian rhythm of medical residents. Mean with 95% CI of the A) amplitude, B) percentage of rhythmicity, C) mesor and D) acrophase of the temperature rhythm (No medical error group: n=11; Medical error group: n=14). Supplementary table 1. Characteristics of the participants that did not complete the medical error subsection (excluded). Supplementary table 2. Bivariate correlation between working characteristics, sleep habits and psyco-affective variables. Supplementary table 3. Demographic, working, sleep and psycho-affective characteristics of the objective study sample. Supplementary table 4. Bivariate correlation between objective working and sleep characteristics, and sleep and psyco-affective scales. [file 12889_2023_17130_MOESM1_ESM.docx]

**Supplementary material**

Index

[Supplementary methods 1](#_Toc137214873)

[Peripheral temperature 1](#_Toc137214874)

[Data analysis 1](#_Toc137214875)

[Supplementary Figures 2](#_Toc137214876)

[Supplementary Figure 1. 2](#_Toc137214877)

[Supplementary Tables 3](#_Toc137214878)

[Supplementary table 1 3](#_Toc137214879)

[Supplementary table 2 4](#_Toc137214880)

[Supplementary table 3 5](#_Toc137214881)

[Supplementary table 4 6](#_Toc137214882)

[References 6](#_Toc137214883)

# Supplementary methods

## Peripheral temperature

Corporal peripheral temperature rhythm was assessed using a 16 mm X 6 mm temperature sensor (Thermochron iButton DS1291H, Dallas Maxim) placed next to the actigraph. Distal skin temperature is considered as a proxy for the circadian rhythm of core temperature; both measures are similar with an approximate 12-h phase difference between them (distal skin temperature reaches its maximum levels during the sleeping period)^1^. The original sample was reduced to 25 individuals (Figure 1) who were the ones that had a recording of almost 3 days and complete the medical error subsection of the questionaire. They were divided into two groups based on their medical error reporting: the no medical error group (n=11) and the medical error group (n=14). Cosinor was calculated using the library “CATkit” from R software and we obtained the following measures: amplitude (difference between the maximum and the midline-estimating statistic of rhythm, % rhythm (percent of the variance explained by the model), acrophase of the rhythm (time at which the maximum of the rhythm occurs), and mesor (midline-estimating statistic of rhythm or the rhythm-adjusted mean).

## Data analysis

Variables derived from cosinor analysis of peripheral temperature rhythm are shown as mean with 95% CI, normality was tested using Kolmogorov-Smirnov test, and groups were compared by means of an unpaired t-test with Welch’s correction for independent samples.

# Supplementary Figures


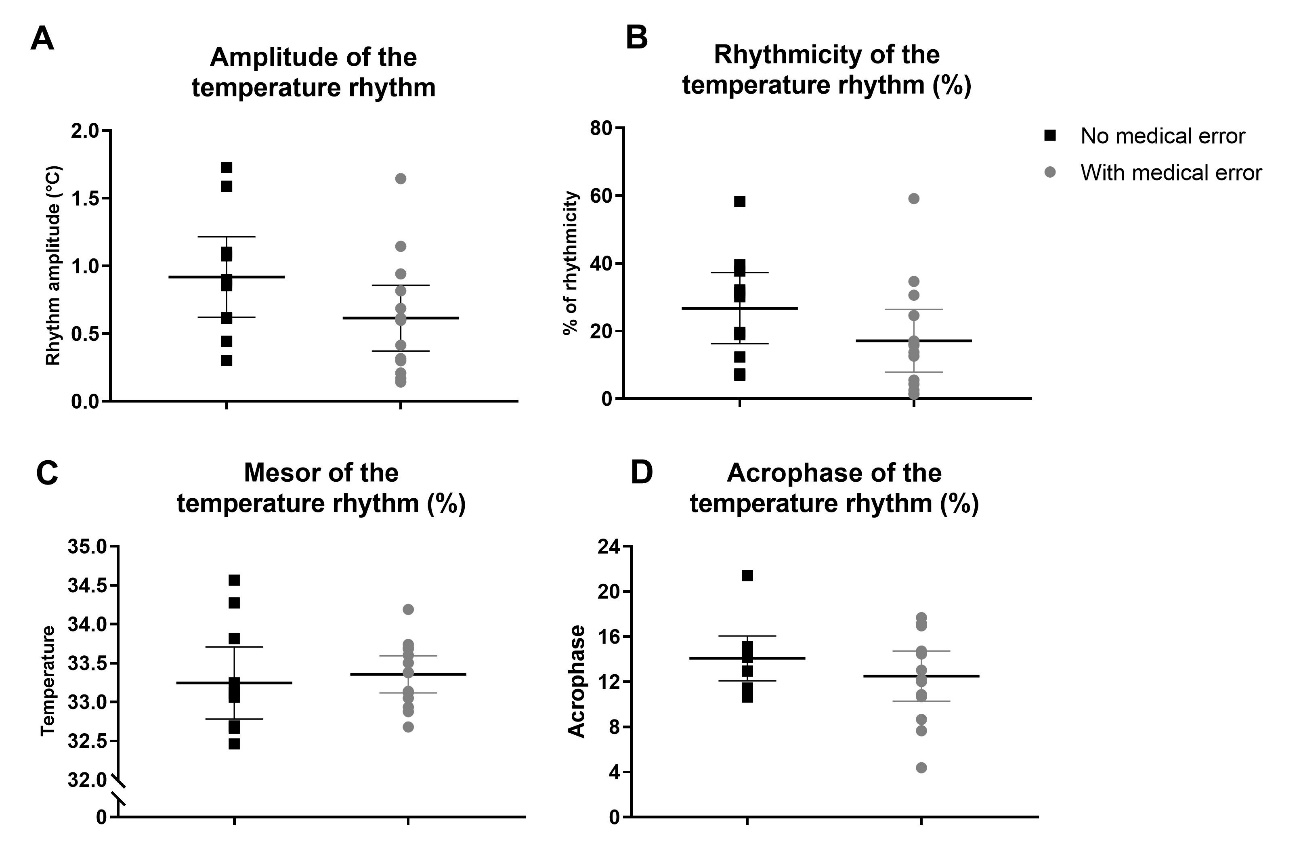


Supplementary Figure 1. Characteristics of the temperature circadian rhythm of medical residents. Mean with 95% CI of the A) amplitude, B) percentage of rhythmicity, C) mesor and D) acrophase of the temperature rhythm (No medical error group: n=11; Medical error group: n=14).

# Supplementary Tables

| *Supplementary table 1* – Characteristics of the participants that did not complete the medical error subsection (excluded) | | | | | | | |
| --- | --- | --- | --- | --- | --- | --- | --- |
| Variable | *Included* | | | *Excluded* | | | *Test* |
|  | *Valid n* | *Mean/n* | *SD/%* | *Valid n* | *Mean/n* | *SD/%* | *p* |
| **Mean age** | 433 | 28.0 | 2.3 | 224 | 28.6 | 3.2 | ***0.006** |
| **Year of residency program** | 436 | 2.4 | 1.1 | 225 | 2.2 | 1.1 | 0.06 |
| **Specialty (internal medicine)** | 444 | 342 | 83.4 | 212 | 158 | 84.5 | 0.57 |
| **Total weekly hours on duty in hospital** | 384 | 66.7 | 21.4 | 182 | 65.2 | 23.0 | 0.46 |
| **Longest continuous shift (hs)** | 389 | 29.3 | 10.3 | 194 | 26.7 | 12.0 | ***0.006** |
| **Monthly on-call shifts** | 414 | 6.0 | 3.0 | 200 | 6.1 | 3.1 | 0.78 |
| **Typical anchor sleep (hs) in a work day** | 433 | 6.0 | 1.6 | 221 | 6.2 | 1.6 | 0.33 |
| **Non-anchor sleep (min) in a work day** | 474 | 21.4 | 57.0 | 253 | 24.7 | 65.0 | 0.49 |
| **Total sleep (hs) in a work day** | 433 | 6.4 | 1.8 | 221 | 6.6 | 2.1 | 0.18 |
| **Sleep debt (min) in workdays** | 425 | 97.3 | 123.0 | 216 | 86.1 | 140.1 | 0.21 |
| **Typical anchor sleep (hs) in free days** | 426 | 9.3 | 2.1 | 220 | 9.3 | 1.8 | 0.95 |
| **Non-anchor sleep (min) in free days** | 474 | 66.0 | 85.8 | 253 | 62.3 | 86.0 | 0.59 |
| **Total sleep (hs) in free days** | 426 | 10.5 | 2.8 | 220 | 10.4 | 2.5 | 0.81 |
| **Sleep debt free-work day (min)** | 424 | 241.6 | 183.6 | 219 | 228.7 | 186.2 | 0.40 |
| **Pittsburgh Sleep Quality Index score** | 436 | 11.7 | 2.2 | 225 | 11.7 | 2.2 | 0.78 |
| **Epworth Sleepiness Scale score** | 436 | 14.4 | 4.9 | 225 | 13.8 | 5.1 | 0.17 |
| **Beck Anxiety Inventory score** | 436 | 9.8 | 8.0 | 225 | 9.0 | 7.8 | 0.21 |
| **Beck Depression Inventory score** | 436 | 5.2 | 4.0 | 225 | 4.8 | 4.4 | 0.35 |
| **Maslach Emotional Exhaustion score** | 436 | 29.3 | 11.6 | 225 | 26.3 | 12.0 | ***0.001** |
| **Maslach Depersonalization score** | 436 | 10.5 | 7.3 | 225 | 8.4 | 7.2 | ***0.001** |
| **Maslach Personal Acomplishment score** | 436 | 35.2 | 8.6 | 225 | 34.2 | 11.0 | 0.207 |

Mean is reported for numerical and percentage for categorical data.

*Significant difference in the independent sample t-test (for numerical variables) and in the Chi-square test (for categorical variables).

| *Supplementary table 2* **–** Bivariate correlation between working characteristics, sleep habits and  psyco-affective variables | | | | | | | | | | |
| --- | --- | --- | --- | --- | --- | --- | --- | --- | --- | --- |
| Variable | | *PSQI score* | *ESS score* | *BAI score* | *BDI score* | *MEE score* | *MDP score* | *MPA score* | *Longest cont. shift* | *Monthly active guards* |
| **Monthly on-call shifts** | *r* | 0115 | 0.191 | 0.025 | 0.103 | 0.137 | 0.147 | 0.015 | 0.409 | 1 |
|  | *p* | ***0.004** | ***<0.001** | 0.530 | ***0.011** | ***<0.001** | ***<0.001** | 0.717 | ***<0.001** | **-** |
| **Longest continuous shift** | *r* | 0.088 | 0.141 | 0.039 | 0.054 | 0.133 | 0.132 | -0.015 | 1 | 0.409 |
|  | *p* | ***0.034** | ***<0.001** | 0.344 | 0.190 | ***0.001** | ***0.001** | 0.721 | - | ***<0.001** |
| **Typical anchor sleep in a work day** | *r* | -0.159 | -0.172 | -0.092 | -0.169 | -0.144 | -0.147 | 0.085 | -0.104 | -0.196 |
|  | *p* | ***<0.001** | ***<0.001** | ***0.018** | ***<0.001** | ***<0.001** | ***<0.001** | ***0.029** | ***0.013** | ***<0.001** |
| **% non-anchor sleep in a work day** | *r* | -0.019 | -0.076 | 0.000 | -0.041 | -0.009 | 0.096 | -0.022 | -0.038 | 0.059 |
|  | *p* | 0.629 | 0.050 | 0.993 | 0.289 | 0.809 | ***0.013** | 0.578 | 0.361 | 0.141 |
| **Total sleep in work days** | *r* | -0.136 | -0.188 | -0.064 | -0.159 | -0.117 | -0.067 | 0.057 | -0.106 | -0.131 |
|  | *p* | ***<0.001** | ***<0.001** | 0.104 | ***<0.001** | ***0.003** | 0.088 | 0.146 | ***0.011** | ***0.001** |
| **Total sleep in free days** | *r* | -0.039 | 0.129 | 0.032 | 0.002 | 0.076 | 0.079 | -0.031 | 0.078 | 0.087 |
|  | *p* | 0.328 | ***0.001** | 0.410 | 0.958 | 0.054 | ***0.044** | 0.435 | 0.063 | ***0.034** |
| **Sleep debt (with total sleep) in work days** | *r* | 0.089 | 0.242 | 0.068 | 0.192 | 0.151 | 0.082 | -0.073 | 0.092 | 0.142 |
|  | *p* | ***0.024** | ***<0.001** | 0.084 | ***<0.001** | ***<0.001** | ***0.038** | 0.065 | ***0.028** | ***<0.001** |
| **Sleep debt free-work days (with total sleep)** | *r* | 0.012 | 0.184 | 0.063 | 0.111 | 0.100 | 0.081 | -0.037 | 0.071 | 0.119 |
|  | *p* | 0.752 | ***<0.001** | 0.111 | ***0.005** | ***0.011** | ***0.039** | 0.354 | 0.089 | ***0.004** |

PSQI, Pittsburgh Sleep Quality Index; ESS, Epworth Sleepiness Scale; BAI, Beck Anxiety Inventory; BDI, Beck Depression Inventory; MEE, Maslach Emotional Exhaustion; MDP, Maslach Depersonalization; MPA, Maslach Personal Accomplishment.

Total n was 661.

r, Pearson’s correlation coefficient

*Significant p-value for the Pearson Correlation analysis.

| *Supplementary table 3* **-** Demographic, working, sleep and psycho-affective characteristics of the objective study sample | | | |
| --- | --- | --- | --- |
| Variable | *Valid n* | *Mean/n* | *SD/%* |
| **Mean age** | 38 | 27.7 | 2.0 |
| **Year of residency program** | 38 | 2.5 | 1.9 |
| **Specialty (internal medicine)** | 35 | 33 | 100 |
| **Total weekly hours on duty in hospital** | 34 | 76.4 | 20.8 |
| **Longest continuous shift (hs)** | 34 | 32.3 | 8.1 |
| **Monthly on-call shifts** | 35 | 7.0 | 2.2 |
| **Typical anchor sleep (hs) in a work day** | 37 | 6.2 | 1.3 |
| **Non-anchor sleep (min) in a work day** | 38 | 15 | 34.4 |
| **Total sleep (hs) in a work day** | 37 | 6.5 | 1.4 |
| **Sleep debt (min) in workdays** | 36 | 91.7 | 74.6 |
| **Typical anchor sleep (hs) in free days** | 38 | 9.8 | 2.0 |
| **Non-anchor sleep (min) in free days** | 38 | 63.9 | 81.4 |
| **Total sleep (hs) in free days** | 38 | 10.8 | 2.6 |
| **Sleep debt free-work day (min)** | 37 | 265.1 | 157.0 |
| **Pittsburgh Sleep Quality Index score** | 38 | 11.9 | 2.0 |
| **Epworth Sleepiness Scale score** | 38 | 14.8 | 5.3 |
| **Beck Anxiety Inventory score** | 38 | 9.9 | 8.3 |
| **Beck Depression Inventory score** | 38 | 5.7 | 3.0 |
| **Maslach Emotional Exhaustion score** | 38 | 32.3 | 12.6 |
| **Maslach Depersonalization score** | 38 | 12.3 | 7.5 |
| **Maslach Personal Acomplishment score** | 38 | 36.1 | 9.5 |

Mean is reported for numerical and percentage for categorical data.

| *Supplementary table 4* **–** Bivariate correlation between objective working and sleep characteristics, and sleep and psyco-affective scales. | | | | | | | | |  |
| --- | --- | --- | --- | --- | --- | --- | --- | --- | --- |
| Variable | | *PSQI score* | *ESS score* | *BAI score* | *BDI score* | *MEE score* | *MDP score* | *MAP score* | *% of diurnal sleep* |
| **Total weekly hours of extended shift** | *r* | 0.39 | 0.49 | 0.59 | 0.39 | 0.51 | 0.23 | -0.11 | 0.50 |
|  | *p* | 0.07 | ***0.02** | ***0.004** | 0.08 | ***0.02** | 0.31 | 0.62 | ***0.02** |
| **% of diurnal sleep** | *r* | 0.41 | 0.33 | 0.52 | 0.24 | 0.47 | 0.53 | -0.14 | 1 |
|  | *p* | 0.06 | 0.14 | ***0.01** | 0.29 | ***0.03** | ***0.01** | 0.54 |  |

PSQI, Pittsburgh Sleep Quality Index; ESS, Epworth Sleepiness Scale; BAI, Beck Anxiety Inventory; BDI, Beck Depression Inventory; MEE, Maslach Emotional Exhaustion; MDP, Maslach Depersonalization; MPA, Maslach Personal Accomplishment.

Total n was 19.

r, Pearson’s correlation coefficient

*Significant p-value for the Pearson Correlation analysis.

# References

1. Sarabia JA, Rol MA, Mendiola P, Madrid JA. Circadian rhythm of wrist temperature in normal-living subjects. A candidate of new index of the circadian system. *Physiol Behav*. 2008;95(4):570-580. doi:10.1016/j.physbeh.2008.08.005
